# The alarmin IL-1α is a master cytokine in acute lung inflammation induced by silica micro- and nanoparticles

**DOI:** 10.1186/s12989-014-0069-x

**Published:** 2014-12-13

**Authors:** Virginie Rabolli, Anissa Alami Badissi, Raynal Devosse, Francine Uwambayinema, Yousof Yakoub, Mihaly Palmai-Pallag, Astrid Lebrun, Valentin De Gussem, Isabelle Couillin, Bernard Ryffel, Etienne Marbaix, Dominique Lison, François Huaux

**Affiliations:** Louvain centre for Toxicology and Applied Pharmacology (LTAP), Institut de Recherche Expérimentale et Clinique (IREC), Université catholique de Louvain, Brussels, Belgium; University of Orléans, CNRS, UMR7355, INEM, Transgenose Institute, Orléans, France; de Duve Institute, Université catholique de Louvain, Brussels, Belgium; Louvain centre for Toxicology and Applied Pharmacology (LTAP), Université catholique de Louvain (UCL), Avenue Mounier 52, B1.52.12, 1200 Brussels, Belgium

**Keywords:** Alarmins, Inflammation, Neutrophils, Silica, Nanoparticles, IL-1 family, Inflammasome

## Abstract

**Background:**

Inflammasome-activated IL-1β plays a major role in lung neutrophilic inflammation induced by inhaled silica. However, the exact mechanisms that contribute to the initial production of precursor IL-1β (pro-IL-1β) are still unclear. Here, we assessed the implication of alarmins (IL-1α, IL-33 and HMGB1) in the lung response to silica particles and found that IL-1α is a master cytokine that regulates IL-1β expression.

**Methods:**

Pro- and mature IL-1β as well as alarmins were assessed by ELISA, Western Blot or qRT-PCR in macrophage cultures and in mouse lung following nano- and micrometric silica exposure. Implication of these immune mediators in the establishment of lung inflammatory responses to silica was investigated in knock-out mice or after antibody blockade by evaluating pulmonary neutrophil counts, CXCR2 expression and degree of histological injury.

**Results:**

We found that the early release of IL-1α and IL-33, but not HMGB1 in alveolar space preceded the lung expression of pro-IL-1β and neutrophilic inflammation in silica-treated mice. *In vitro*, the production of pro-IL-1β by alveolar macrophages was significantly induced by recombinant IL-1α but not by IL-33. Neutralization or deletion of IL-1α reduced IL-1β production and neutrophil accumulation after silica in mice. Finally, IL-1α released by J774 macrophages after *in vitro* exposure to a range of micro- and nanoparticles of silica was correlated with the degree of lung inflammation induced *in vivo* by these particles.

**Conclusions:**

We demonstrated that in response to silica exposure, IL-1α is rapidly released from pre-existing stocks in alveolar macrophages and promotes subsequent lung inflammation through the stimulation of IL-1β production. Moreover, we demonstrated that *in vitro* IL-1α release from macrophages can be used to predict the acute inflammogenic activity of silica micro- and nanoparticles.

**Electronic supplementary material:**

The online version of this article (doi:10.1186/s12989-014-0069-x) contains supplementary material, which is available to authorized users.

## Background

Inhalation of micrometric and nanometric particles can lead to pulmonary diseases characterized by inflammation, fibrosis and/or cancer. Pulmonary inflammation induced by inhaled particles is characterized by a marked accumulation of neutrophils which secrete large quantities of reactive oxygen metabolites, granule enzymes and pro-inflammatory mediators [1,2]. It has been demonstrated that the pro-inflammatory cytokine interleukin-1β (IL-1β) drives particle-induced inflammation by enhancing granulocyte migration and accumulation to inflammatory sites [3-5]. Mature IL-1β release in the extracellular environment requires several steps including the production of the biologically inactive IL-1β pro-form (pro-IL-1β) consecutive to Il1b gene transcription as well as its maturation and exocytosis through the NLRP3 inflammasome machinery. Pro-IL-1β production could result from cellular detection of pathogen components, PAMPs, or from pro-inflammatory cytokines. Also, IL-1β positively auto-regulates its own synthesis via NFκb binding activity [6].

Several studies have demonstrated that K^+^ efflux, cytosolic release of lysosomal cathepsins and mitochondria-derived factors such as reactive oxygen species (ROS) are involved in the NLRP3 inflammasome assembly and activation by micro- and nanoparticles [7-11]. It is, however, surprising that the signal(s) triggering NFκb-mediated up-regulation of pro-IL-1β after particle exposure remain(s) unknown [12,13]. The identification of a common element that initiates pro-IL-1β production in response to particles would be useful to understand their inflammatory properties and to predict their inflammatory potential.

The alarmin family comprises structurally distinct endogenous mediators including defensins, cathelicidins, eosinophil-associated ribonucleases, heat shock proteins (HSP), saposin-like granulysin, ion-binding proteins (e.g., S100 proteins and lactoferrin), and nucleotides/metabolites (e.g., uric acid) [14]. Additionally, some constitutive cytokines that possess intracellular functions such as IL-1α, IL-33 and High Mobility Group Box 1 (HMGB1) are also considered as alarmins [5,15-18]. Alarmins are passively released from necrotic cells upon infection and tissue injury or rapidly secreted by stimulated leukocytes. Alarmins play important intracellular roles in homeostasis and when released extracellularly strongly promote leukocyte cell recruitment and activation as well as tissue repair. This occurs for instance in response to sterile injury or infection [19].

IL-1α and IL-33 are produced as pro-form but contrary to IL-1β, these precursors are already active. Upon cell necrosis, the released IL-1α, IL-33 and HMGB1 alarmins alone or complexed to other molecules bind their respective receptors (IL-1R1, ST2 and RAGE, TLR2 or TLR4) and promote pro-inflammatory gene transcription via the NFκb pathway [16]. We thus hypothesized that lung injury induced by toxic particles leads to the release of intracellular alarmins which can induce pro-IL-1β production and subsequent inflammatory processes.

In this study, we identified the initial events leading to the lung inflammatory response to inhaled particles and found that the constitutively available alarmin IL-1α is rapidly released in alveolar space in response to silica exposure, before IL-1β induction and pulmonary neutrophil influx. We demonstrated that IL-1α can directly induce pro-IL-1β production by alveolar macrophages and that its neutralization impairs silica-induced lung IL-1β release and inflammation. Finally, we identified macrophages as a main source of IL-1α in the lung and developed an *in vitro* assay to evaluate the inflammogenic activity of nano- and micrometric particles based on their capacity to release IL-1α from macrophages.

## Results

### The early release of the endogenous IL-1α and IL-33 alarmins precedes silica-induced IL-1β production and neutrophilic inflammation in mice

**Figure 1:**
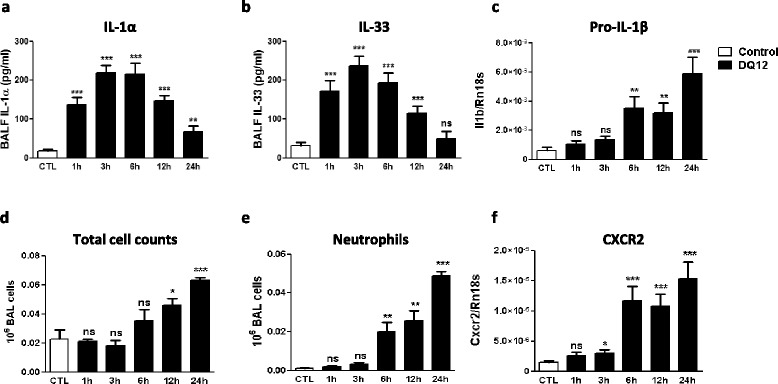
**Silica induces IL-1α and IL-33 release in the lung before IL-1β production and neutrophilic inflammation.** Levels of **(a)** IL-1α and **(b)** IL-33 in BAL fluid collected at different time points after silica (crystalline DQ12, 2.5 mg) or not (control). Pulmonary expression of **(c)** pro-IL-1β quantified by qRT-PCR at different time points after instillation of silica or not. Number of alveolar **(d)** total cells and **(e)** neutrophils (GR1^+^ cells) assessed by flow cytometry. **(f)** Expression of the pulmonary neutrophilic CXCR2 marker quantified by qRT-PCR at different time points after silica or not. Values are means ± SEM of 3 to 8 animals. *p < 0.05, **p < 0.01 and ***p < 0.001 denote significant difference between animals treated with silica or not; ns, denotes no significant difference. P-values are estimated by *t*-test.

In order to explore the implication of alarmins in particle-induced IL-1β production in the lung, we first measured in broncho-alveolar lavage fluid (BALF) and lung tissue the protein and gene expression of IL-1α, IL-33 and HMGB1 at different time points after an inflammatory dose of micrometric crystalline silica (DQ12, 2.5 mg) [20,21]. One hour after silica administration, IL-1α and IL-33 protein levels were already significantly increased in BALF. This release peaked at 6 and 12 hours and progressively returned to control values at 24 hours (Figure 1a and b). Silica did not affect BALF HMGB1 levels (Additional file 1: Figure S1a). An increase of lung IL-1α, IL-33 and HMGB1 transcript contents was only observed from 6 hours after silica administration and this effect was maintained up to 24 hours (Additional file 1: Figure S1d, e and f). These data suggest that preexisting stocks of IL-1α and IL-33 protein are rapidly released in the lung after silica.

The early lung release (1 h) of IL-1α and IL-33 after silica preceded the increased expression of pro-IL-1β and the release of mature IL-1β. Indeed, the levels of lung IL-1β transcripts (Figure 1c) and BALF IL-1β protein (Additional file 1: Figure S1b) were mainly increased between 6 and 24 hours following instillation. Cellular lung inflammation was first monitored by BAL total cell and neutrophil (GR1+ cells) counts. Neutrophil accumulation was also quantified by assessing lung expression of CXCR2. Although the expression of this chemokine receptor has been reported in macrophages, CXCR2 is mainly expressed by recruited neutrophils and can be used as a biomarker of neutrophilic inflammation [22]. Akin biochemical parameters (Additional file 1: Figure S1c), cellular inflammation was obvious 6 hours after silica and persisted until 24 hours (Figure 1d to f). These data suggested that the rapid release of the intracellular stocks of IL-1α and IL-33 contributes to IL-1β production and neutrophilic inflammation following silica exposure.

### The alarmin IL-1α induces pro-IL-1β production in alveolar macrophages

**Figure 2:**
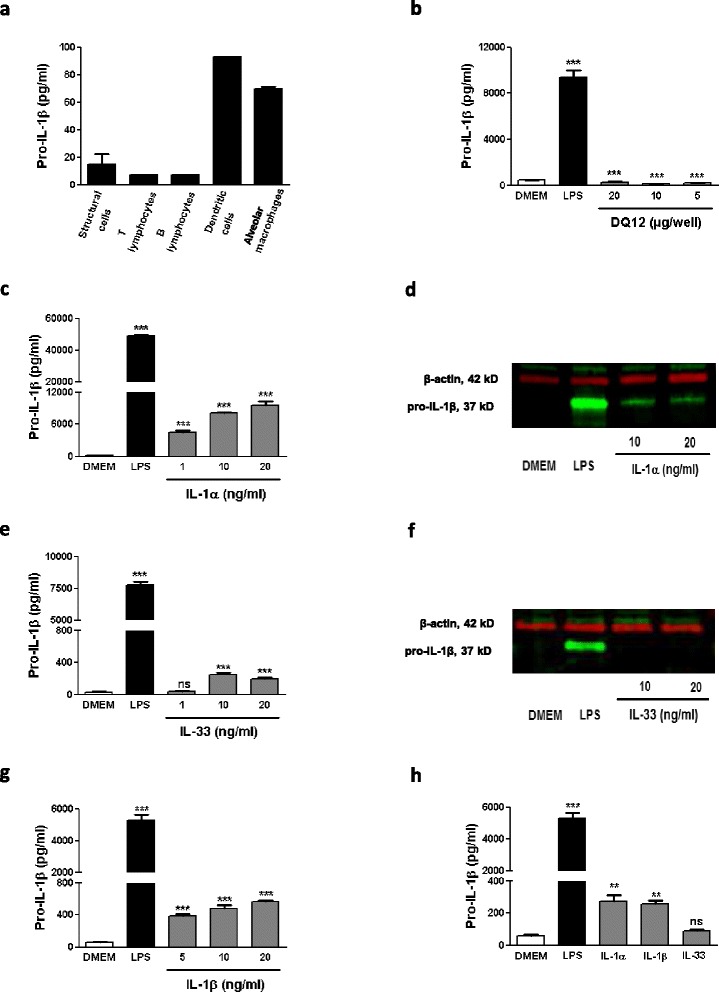
**IL-1-induced pro-IL-1β production in alveolar macrophages. (a)** Intracellular levels of pro-IL-1β in structural cells (CD45^-^ cells), T (CD5^+^ cells) and B (B220^+^ cells) lymphocytes, dendritic cells (F4/80^-^ CD11c^+^ cells) and alveolar macrophages (F4/80^+^) purified from lungs 3 hours after silica instillation (crystalline DQ12, 2.5 mg). n = 2 to 6. **(b)** Intracellular levels of pro-IL-1β in primary cultured alveolar macrophages exposed overnight to LPS (0.1 μg/ml) or silica (DQ12). n = 4. Intracellular levels of pro-IL-1β in primary cultured lung macrophages exposed overnight to LPS (0.1 μg/ml) or recombinant IL-1α, IL-33 or IL-1β in different experiments (**(c)**, **(e)** and **(g)** respectively) or in the same experiment (10 ng/ml) **(h)**. n = 3 to 5. Western blot analysis of intracellular pro-IL-1β and β-actin in primary cultured alveolar macrophages exposed during 18 hours to LPS (0.1 μg/ml), **(d)** recombinant IL-1α or **(f)** IL-33. Values are means ± SEM. **p < 0.01 and ***p < 0.001 denotes significant difference between cells in DMEM and cells exposed to LPS, silica, recombinant IL-1α, recombinant IL-33 or recombinant IL-1β; ns, denotes no significant difference. P-values are estimated by *t*-test.

We next tested whether the alarmins IL-1α and IL-33 can directly activate the *in vitro* expression of pro-IL-1β. First, we determined the main cellular source of IL-1β in the lung of mice following silica exposure. IL-1β production is well defined in immune cells but other sources such as epithelial cells have been recently identified [23,24]. Therefore, we purified structural (epithelial cells and fibroblasts) and immune cells (i.e. T and B lymphocytes, dendritic cells and macrophages) from the lung of silica-treated mice and measured their pro-IL-1β intracellular contents. Lymphocytes and structural cells produced little amount of pro-IL-1β after silica exposure. Alveolar macrophages and dendritic cells produced high levels of pro-IL-1β and were the major cell populations expressing IL-1β in silica treated mice (Figure 2a). We also verified that silica alone did not immediately stimulate pro-IL-1β synthesis in primary lung macrophage cultures (Figure 2b). Interestingly, recombinant IL-1α induced a dose-dependent pro-IL-1β production by alveolar macrophages as appreciated by ELISA (Figure 2c) and western blot analysis (Figure 2d). After recombinant IL-33 addition, a slight but not dose-dependent increase of pro-IL-1β levels was observed by ELISA (Figure 2e) but not by WB analysis (Figure 2f). As expected, the addition of recombinant mature IL-1β in macrophage cultures dose-dependently induced the expression of its pro-form (Figure 2g). At the same concentration, recombinant IL-1α and IL-1β but not IL-33 induced similar production of pro-IL-1β by alveolar macrophages (Figure 2h). Several forms of recombinant HMGB1 [25,26] had no effect on pro-IL-1β expression when added to macrophages (data not shown). Altogether, these results indicated that the alarmin IL-1α and mature IL-1β strongly stimulate pro-IL-1β production by alveolar macrophages.

### The alarmin IL-1α is necessary for IL-1β production in response to silica particles in the mouse lung

**Figure 3:**
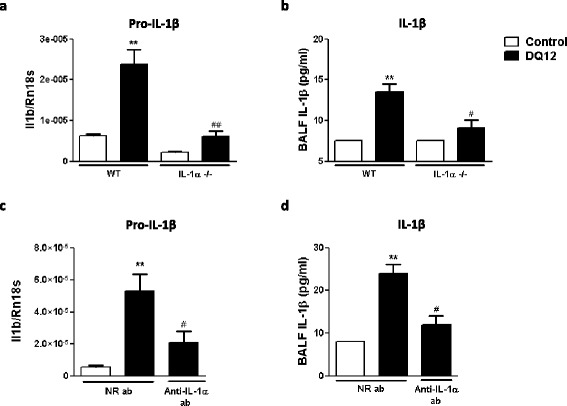
**IL-1α inhibition reduces IL-1β production in lung of silica-treated mice. (a)** Pulmonary expression of pro-IL-1β quantified by qRT-PCR and **(b)** levels of mature IL-1β in BAL fluid of WT and IL-1α KO mice, 24 hours after instillation of silica (crystalline DQ12, 2.5 mg) or not (control). **(c)** Pulmonary expression of pro-IL-1β quantified by qRT-PCR and **(d)** levels of mature IL-1β in BAL fluid 18 hours after silica or not, in mice treated with IL-1α neutralizing antibody or not. Values are means ± SEM of 3 to 8 animals. **p < 0.01 denote significant difference between animals treated or not with silica. # p < 0.05, ## p < 0.01 denote significant difference between silica-treated animals with decreased or unmodified IL-1α. P-values are estimated by *t*-test.

Because IL-1α release preceded the expression of IL-1β in silica-treated mice (Figure 1a and c), we then delineated the implication of IL-1α on silica-induced pro-IL-1β production and mature IL-1β release by examining the secretion of IL-1β after silica treatment in mice lacking IL-1α. The genetic absence of IL-1α abrogated pro- and mature IL-1β expression (Figure 3a and b) in response to silica. Injection of neutralizing antibodies directed against IL-1α [27,28] also markedly decreased the levels of IL-1β transcript (Figure 3c) and protein (Figure 3d) in response to silica. These data strongly indicated that the rapid release of IL-1α promotes IL-1β production in the lung of silica-treated mice.

### The alarmin IL-1α contributes to neutrophilic inflammation in response to silica particles in the lung of mice

**Figure 4:**
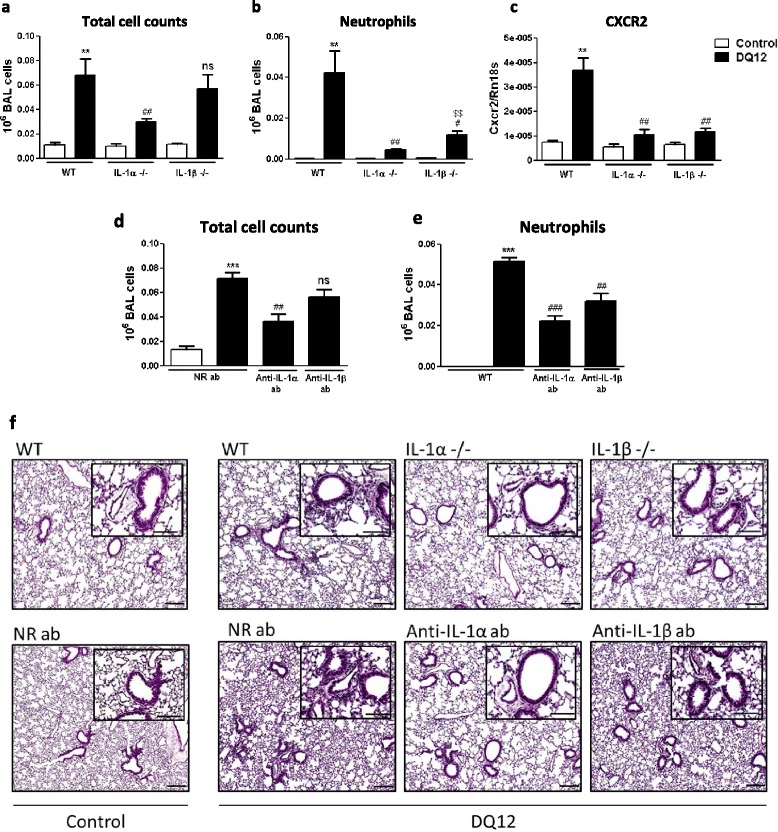
**IL-1α and IL-1β inhibition reduces neutrophilic inflammation in lung of silica-treated mice. (a)** Number of alveolar total cells in WT, IL-1α KO or IL-1β KO mice 24 hours after instillation of silica (crystalline DQ12, 2.5 mg) or not (control). **(b)** Number of alveolar neutrophils (GR1+ cells) assessed by flow cytometry and **(c)** expression of the pulmonary neutrophilic CXCR2 marker quantified by qRT-PCR in WT, IL-1α KO or IL-1β KO mice 24 hours after instillation of silica or not. Number of alveolar **(d)** total cells and **(e)** neutrophils in WT mice treated with anti-IL-1α or IL-1β neutralizing antibodies or not 18 hours after silica or not. **(f)** Hematoxylin and eosin-stained lung sections obtained from untreated WT mice or after silica instillation of WT, IL-1α KO and IL-1β KO mice or from untreated mice or after silica instillation of mice injected or not with anti-IL-1α or anti-IL-1β antibodies. Scale bars = 200 μm (large panels) and 100 μm (inserts). Values are means ± SEM of 3 to 5 animals. **p < 0.01 and ***p < 0.001 denote significant difference between animals treated with silica or not. # p < 0.05, ## p < 0.01 and ### p < 0.001 denote significant difference between silica-treated animals with decreased or unmodified IL-1α or IL-1β activity. $$ p < 0.01 denote significant difference in values between IL-1α KO and IL-1β KO silica-treated animals. Ns, denotes no significant difference between silica-treated animals with decreased or unmodified IL-1α or IL-1β activity. P-values are estimated by *t*-test.

We next determined the role of IL-1α in the development of lung inflammation induced by silica by examining neutrophil accumulation. First, we confirmed the pivotal role of IL-1β in lung inflammation development since neutrophil influx after silica was significantly impaired after IL-1β inactivation (KO and blocking antibodies, Figure 4a-f). Genetic deletion of Il1a resulted in a deeper and more complete inhibition of cellular inflammation as appreciated by inflammatory cell and neutrophil accumulation (Figure 4a and b), CXCR2 expression (Figure 4c) as well as histological analyses (Figure 4f). Antibody inactivation confirmed the major implication of IL-1α in silica-induced neutrophilic inflammation (Figure 4d-f). Altogether, these data indicated that the alarmin IL-1α indirectly promotes particle-induced lung inflammation through the induction of IL-1β production but also possesses a direct inflammatory activity.

### The *in vitro* release of IL-1α by macrophages upon particle exposure predicts the *in vivo* inflammatory activity of the particles

**Figure 5:**
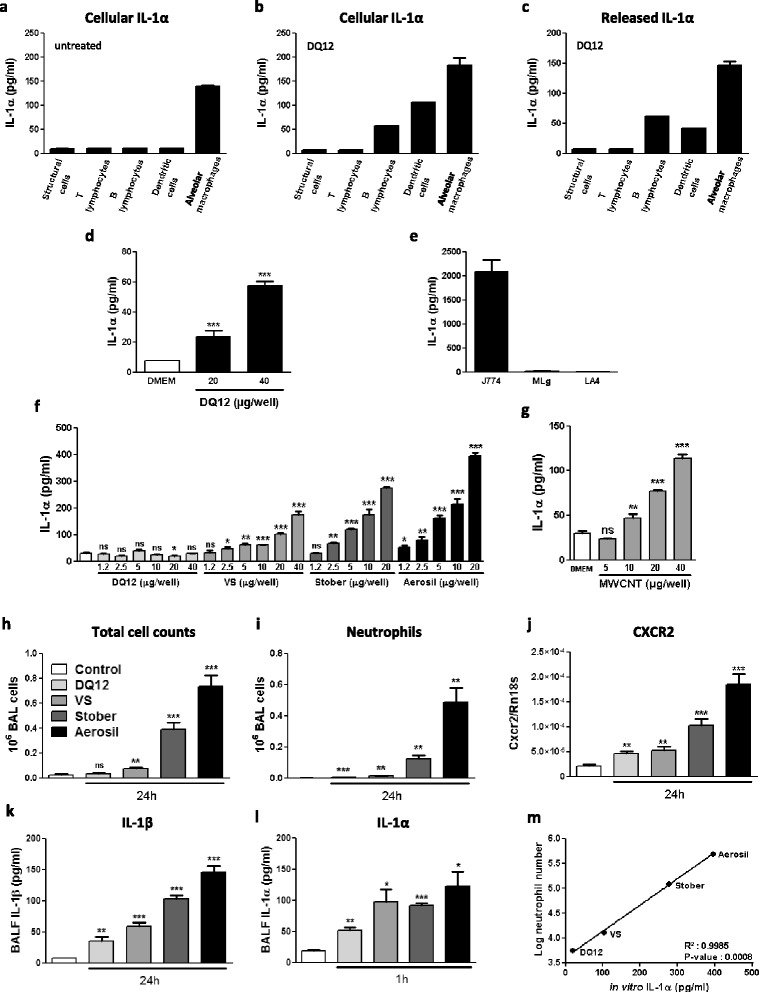
**IL-1α release by J774 macrophages after particle exposure predicts acute lung inflammation upon particle treatment in mice.** Intracellular levels of IL-1α in structural cells (CD45^-^ cells), T (CD5^+^ cells) and B (B220^+^ cells) lymphocytes, dendritic cells (F4/80^-^ CD11c^+^ cells) and alveolar macrophages (F4/80^+^) freshly purified from **(a)** untreated lung or **(b)** 3 hours after silica instillation (crystaline DQ12, 2.5 mg). n = 1 to 6. **(c)** Release of IL-1α by structural cells (CD45^-^ cells), T (CD5^+^ cells) and B (B220^+^ cells) lymphocytes, dendritic cells (F4/80^-^ CD11c^+^ cells) and alveolar macrophages (F4/80^+^) purified from lungs 3 hours after silica instillation. n = 1 to 6 **(d)** Release of IL-1α by fresh primary lung macrophages (CD45^+^ F4/80^+^) exposed to silica (overnight). n = 4. **(e)** Intracellular levels of IL-1α in macrophage (J774), fibroblast (MLg) and epithelial (LA4) cell line. n = 2 to 6. **(f)** Release of IL-1α by J774 exposed overnight to increasing concentration of DQ12, Vitreous Silica (VS), Stöber silica nanoparticles (Stober) and Aerosil 200. n =3 to 4. **(g)** Release of IL-1α by J774 exposed overnight to various concentration of MWCNT. n =3 to 4. **(h)** Number of alveolar total cells 24 hours after particle instillation (1.2 mg) or not. **(i)** Number of alveolar neutrophils (GR1^+^ cells) assessed by flow cytometry and **(j)** expression of the pulmonary neutrophilic marker CXCR2 quantified by qRT-PCR in mice 24 hours after particle instillation or not. Levels of **(k)** mature IL-1β or **(l)** IL-1α in BAL fluid collected respectively 24 hours or 1 hour after instillation of particles or not. **(m)** Correlation between alveolar neutrophils (log GR1^+^ cells) collected 24 hours after particle instillation and levels of IL-1α in supernatant of particle-exposed J774. Values are means ± SEM of 3 to 6 animals. *p < 0.05, **p < 0.01 and ***p < 0.001 denote significant difference between particle-treated and not animals; ns, denotes no significant difference. P-values are estimated by *t*-test. Correlation is calculated based on Pearson coefficient.

*In vivo*, IL-1α release upon silica exposure was strongly associated with the subsequent lung inflammatory response. We thus wondered whether the capacity of particles to induce IL-1α cell release determines their inflammatory activity and whether it can be used *in vitro* to predict their toxicity. To test this hypothesis, we first determined the cellular source of the alarmin IL-1α and assessed *in vivo* and *in vitro* its release in response to particles. Intracellular IL-1α content was measured in different structural and immune lung cell populations from naïve or silica-treated mice. Among pulmonary resident cells, only macrophages contained significant amounts of IL-1α in control animals (Figure 5a). After silica treatment, alveolar macrophages contained and released high amount of IL-1α (Figure 5b and c). To a lesser extent, dendritic cells and B lymphocytes also produced and released IL-1α after silica instillation. Moreover, freshly purified alveolar macrophages from naïve mice released IL-1α in the medium in response to silica exposure (Figure 5d). These data indicated that alveolar macrophages represent the main IL-1α-secreting lung cells and can be used to assess the alarmin IL-1α release in response to silica *in vitro*.

In order to develop an *in vitro* screening test, we next assessed the intracellular IL-1α content in murine macrophage (J774), fibroblast (MLg) and epithelial (LA-4) cell lines. Results on cell lines confirmed observations made on primary lung cells, i.e. that macrophages possess high constitutive IL-1α content, contrary to fibroblastic and epithelial cells (Figure 5e). We then compared IL-1α released by J774 in response to increasing amounts of different silica particles: micrometric crystalline silica (DQ12), micrometric amorphous silica (Vitreous Silica; VS), and two different amorphous nanosilica (Stöber and Aerosil 200) (Figure 5f). While DQ12 silica induced a slight release of IL-1α in J774 macrophage supernatant, vitreous and Stöber silica induced a significant IL-1α release from 2.5 μg of particle per well, while Stöber-induced IL-1α release was higher. Finally, Aerosil 200 already induced a significant increase of IL-1α levels at the dose of 1.2 μg/well. Based on these data, we concluded that the tested silica particles induce IL-1α release by J774 macrophages in the following order: DQ12 < VS < Stöber < Aerosil 200. In parallel, mice were instilled with 1.2 mg of the 4 silica particles and lung IL-1α release, neutrophilic accumulation and IL-1β expression were evaluated. IL-1α concentrations assessed in BALF 1 hour after particle instillation were increased as *in vitro*: DQ12 induced a limited release of IL-1α, VS and Stöber were associated with a moderate release of IL-1α while Aerosil induced the strongest IL-1α release (Figure 5l). Particle-induced inflammatory and neutrophilic accumulation (Figure 5h-j) and IL-1β production (Figure 5k) also correlated to *in vitro* IL-1α release. Correlation analyses between *in vitro* IL-1α release (20 μg of particle/well) and *in vivo* neutrophilic accumulation induced by the different silica particles confirmed the clear association between the *in vivo* lung inflammation intensity and the IL-1α release (Figure 5m, R^2^ = 0.9985, p-value 0.0008). MWCNT, known for their lung inflammatory potential [29], also induced IL-1α release from macrophages (Figure 5g). In conclusion, IL-1α release by J774 macrophages was predictive of the acute lung inflammation consecutive to silica particle exposure.

## Discussion

Recent evidence demonstrated that NLRP3 inflammasome activation by particles is pivotal in the release of the pro-inflammatory cytokine IL-1β and the development of lung inflammation [4,7,11]. Particles such as silica activate the inflammasome platform through potassium-, cathepsin- and/or ROS-dependent pathways and allow the release of the cleaved and active IL-1β. The early events leading to pro-IL-1β production following exposure to particles are, however, not identified yet. In models of sterile inflammation, damaged and necrotic cells release intracellular stores of biologically active alarmins that initiate subsequent pro-inflammatory cytokine transcription and neutrophil recruitment [30,31]. We thus postulated that early alarmin release due to particle-induced cell damage can induce pro-IL-1β production and subsequent inflammatory reaction. We discovered that IL-1α and IL-33 were released in response to particle instillation from preexisting cellular stocks. To our knowledge, this is the first time that early release of these alarmins is evaluated in the genesis of inflammation induced by inorganic particles.

Once in the extracellular environment, IL-1β and IL-1α both bind to the IL-1R1/IL-1rAcP complex and induce similar effects. However, contrary to IL-1β, IL-1α does not require maturation and/or exocytosis to be active; its precursors form can activate IL-1R1 or translocate to the nucleus where it will modify inflammatory gene transcription [32]. The role of the IL-1 family in particle-induced neutrophilic inflammation has been extensively investigated mainly by using IL-1 signaling pathway inhibition [3,11] i.e. IL-1Ra (IL-1receptor antagonist) administration [33] or inflammasome activation impairment [3,7,34]. Due to their functional redundancy, these studies do not allow to discriminate between IL-1α or IL-1β implication. Some reports specifically demonstrated a tempering effect of IL-1β deficiency on particle-induced inflammation [4,5,35,36]. A dominant role of IL-1α compared to IL-1β has been demonstrated in nano-TiO2-induced peritonitis and lung neutrophilic inflammation by using IL-1R-, IL-1α- or inflammasome component-deficient mice [23]. In the present study, we newly demonstrate the implication of IL-1α in pro-IL-1β production consecutive to particle exposure. Indeed, IL-1α stimulated precursor IL-1β production by macrophages and the absence of this alarmin abrogated mature IL-1β release upon silica treatment in mice. This is in accordance with observations of Gross and co-authors who reported that absence of IL-1α reduced IL-1β levels measured in response to monosodium urate (MSU) intraperitoneal injection [5]. Similarly, IL-1α neutralizing antibodies decreased lung IL-1β induction due to cigarette smoke [37]. The effect of IL-1α on IL-1β induction was also directly observed on macrophages *in vitro* [5,15,32,38-40]. Interestingly, the implication of IL-1α as a dominant IL-1 cytokine has also been recorded in other murine models of toxic lung inflammation or peritonitis. Alveolar and peritoneal neutrophilic accumulation induced by bleomycin, cigarette smoke or MSU were reduced when IL-1α response was inhibited [5,27,37,41].

IL-1α is a constitutive and ubiquitary cytokine (reviewed in [32]) whose release is frequently assessed in epithelial cells [41,42] or macrophages [15,43,44]. In this paper, we established that among lung resident cells, macrophages represent a major source of available IL-1α. We also demonstrated their ability to release IL-1α in response to particle exposure. Resident macrophages have already been implicated in IL-1α release under inflammatory conditions in lung [37], but also in liver [45] and brain [46]. It has been recently shown that particles-induced pyroptosis, a lytic mode of cell death that exhibits cytoplasmic swelling and ruptures of the plasma membrane [47]. It is tempting to postulate that pyroptosis results in the release of IL-1α by dying macrophages. In addition, it is surprising to note that the release of IL-1α by alveolar macrophages exposed *in vitro* to silica particles does not result in a direct production of pro-IL-1β. IL-1α-releasing macrophages are dying cells and thus probably unable to newly produce pro-IL-1β. In vivo, extracellular IL-1α from necrotic macrophages could, however, trigger pro-IL-1β production by surrounding active macrophages which then propagate neutrophilic inflammation by processing IL-1β after silica endocytosis. Finally, amorphous silica particles are generally regarded as less harmful than the crystalline forms [48-50]. We showed that amorphous (nano)silica particles induced stronger IL-1α secretion and inflammation than crystalline particles. These observations are in line with the recent conclusions offered by several experimental studies indicating that some amorphous (nano)silica particles need to be considered as potent toxic entities [51-55].

We evidenced that IL-33 is released in the lung upon particle instillation. This alarmin did, however, not participate significantly in IL-1β production by lung cells. We also observed a strong induction of IL-33 mRNA 24 hours after silica instillation, suggesting a possible implication in later inflammatory processes. Indeed, recent studies have shown that IL-33 is released in response to particle administration [56,57] and is implicated in lung inflammation after 30 days [58-60].

As observed after cristobalite or asbestos exposure [24,33], it has been recently found that the alarmin HMGB1 was released in alveolar space after MWCNT treatment in mice [61]. The secreted HMGB1 enhanced IL-1β release from alveolar macrophages and its neutralization reduced lung IL-1β content and inflammation *in vivo* [61]. In contrast, we did not observe an increase of HMGB1 alveolar levels in response to silica and different recombinant forms of HMGB1 did not stimulate IL-1β production by alveolar macrophages *in vitro*. This apparent discrepancy could be explained by the type of pulmonary cells targeted by the particles. MWCNT may cause strong cell damage to epithelial cells while silica may preferentially affect macrophages. This could result in the activation of different endogenous danger signal and inflammatory pathways, i.e. related to HMGB1 or IL-1α, respectively. Importantly, Jessop and colleagues demonstrated that HMGB1 present in BAL fluid of MWNCT-treated mice induced IL-1β release by macrophages while recombinant failed. This suggest that the complexation with other inflammatory molecules [62] or the redox status of HMGB1 [25] are mandatory for HMGB1 inflammatory activity. These modifications may also prevent HMGB1 recognition by the ELISA assays and interfere with HMGB1 detection after particle treatment.

The activation of the NFkB pathway leading to il-1b gene expression is probably not limited to IL-1α. Indeed, the pro-inflammatory cytokine TNF-α is also known to induce NFkB activation and IL-1β production [33,63]. Also this cytokine can be released rapidly and independently of transcriptional induction [64]. It is well known that TNF-α is a key factor in the development of lung inflammation upon particle exposure [33,65-67]. As for IL-1α, we showed that TNF-α induced the production of pro-IL-1β in primary lung macrophages *in vitro* (Additional file 1: Figure 2), suggesting that a pleiad of pro-inflammatory mediators may indirectly amplify the production of immature IL-1β. Additional investigations are also needed to better delineate the role the alarmin S100 and heat shock proteins highly secreted following particle exposure [68-70].

Increased levels of IL-1 cytokines were reported in patients suffering from particle-associated inflammatory diseases such as asbestosis [71] and silicosis [72-74]. Targeting IL-1β, e.g. through the use of Anakinra (a recombinant non-glycosylated version of human IL-1Ra) is already in use for the clinical treatment of gout and other IL-1β-related autoimmune diseases [75]. Interestingly, the interaction of Anakinra with the IL-1 receptor prevents the biological functions of IL-1β but also of IL-1α. Our data extend this therapeutic possibility in particle-induced lung inflammation by showing that blocking the IL-1 α/β pathway significantly reduces the deleterious influx of neutrophils after particle exposure. It remains, however, to demonstrate whether IL-1 blockade could also be associated with a reduction of particle-induced lung fibrosis [48]. Indeed, we recently showed that mice lacking IL-1β, IL-1α or IL-1R and treated with DQ12 silica presented a reduction of chronic lung inflammation and granuloma formation compared to their WT counterparts. However, this strong effect on inflammatory development was not concomitantly accompanied by a reduction of lung fibrosis, suggesting that inhibiting IL-1-driven inflammation may not be sufficient to control the fibrotic lung disease after particle exposure [76].

Finally, we showed that IL-1α release by the macrophage J774 cell line is highly predictive of the acute inflammogenic potential of silica micro- and nanoparticles. These results are in agreement with other studies that demonstrated the predictive value of IL-1α release to screen the skin irritative potential of various chemicals [77-80]. Hence, the *in vivo* inflammatory response to silica can be predicted by a simple and quantitative model based on *in vitro* release of IL-1α by cell line macrophages. This model may serve in (nano)toxicology to predict the *in vivo* toxicity of new materials and particles and to reduce animal uses.

## Conclusion

**Figure 6:**
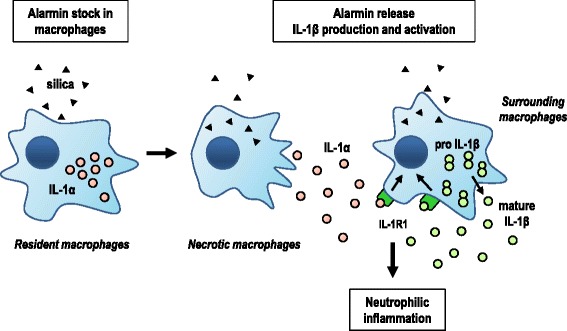
**IL-1α released by alveolar macrophages upon particle exposure mediates neutrophilic inflammation directly and via IL-1β production.** In homeostasis, IL-1α is constitutively expressed by resident alveolar macrophages and intracellularly retained as preexisting stocks. Released IL-1α into the extracellular milieu by necrotic macrophages serves as alarmin after silica exposure. IL-1α is a potent activating stimulus required for surrounding macrophages to express the biologically inactive precursor IL-1β (pro-IL-1β). This form is cleaved by silica-induced inflammasome assembly and activation prior its secretion as mature and bioactive IL-1β. IL-1α and β relayed by their receptor (IL-1R) are necessary to generate pulmonary neutrophil accumulation in response to silica particles.

The present study clarifies the respective role of IL-1α and β in particle-induced lung inflammation. We found that the release of endogenous IL-1α represents an early and crucial event that determines lung inflammatory responses to particle in mice (Figure 6). Released IL-1α after particle exposure serves as an alarmin that triggers the expression and the secretion of IL-1β. Alveolar macrophages represent the major source of IL-1α and IL-1β and macrophage population can be used to develop *in vitro* assays useful for screening the acute inflammatory potential of various types of particles. Finally, our results emphasize the need to target both IL-1α and IL-1β for regulating particle-associated lung inflammatory diseases.

## Materials and methods

### Particles

Crystalline silica particles (DQ12; DMT GmbH and Co. KG, Essen, Germany), Vitreous silica (VS; a gift from Dr. Ghiazza, University of Torino, Italy), Aerosil 200 (Sigma-Aldrich, Bornem, Belgium) and multi-wall carbon nanotubes (MWCNT; MWNT-7, Mitsui, Tokyo, Japan) powders were sterilized by heating at 200°c for 2h immediately before suspension. Sterile Stöber particle suspension were synthesized based on Stöber process and its characterization was described elsewhere [81]. Characteristics of the particles are listed in Table 1.

**Table 1:** **Physicochemical characterization of the particles**

**Name**	**Type**	**Crystallinity**	**Size TEM (nm)**	**Surface BET (m** ^**2**^ **/g)**
DQ12	Ground natural mineral	Crystalline	960^a^	7,4 -10,1^b^
VS	Ground fused silica	Amorphous	1600^c^	3.1^c^
Stöber	Stöber silica	Amorphous	12^d^	400^d^
Aerosil 200	Fumed silica	Amorphous	12^e^	175-225^e^
MWCNT	Multi-wall carbon nanotubes	N/a	Length :5,700 ± 3,700^f^	22^f^
			Width : 74 ± 28^f^	

(a) [82].

(b) [83].

(c) [51].

(d) [84].

(e) Data provided by the manufacturer (Sigma-Aldrich).

(f) [85].

### Animal model

Female C57BL/6 mice were obtained from Janvier (Le Genest-Saint-Isle, France). IL-1α-, IL-1β-, IL1-R1- and MyD88-competent and -deficient mice (in C57BL/6 background) were obtained from the Transgénose Institute (Orleans, France). Studies were performed on gender-matched littermates aged 8-12 weeks. The animals were housed in positive pressure air-conditioned units (25°C, 50% relative humidity) on a 12-hr light/dark cycle and had access to standard diet and tap water ad libitum. The experiments were conducted in accordance with the National Research Council Guide for the Care and Use of Laboratory Animals and approved by the institutional Ethics Committee. Suspensions of crystalline DQ12 silica particles in sterile water or 0.9% saline (Braun Medical, Diegem, Belgium) were injected directly into the lung by pharyngeal aspiration at a dose of 2.5 mg/mouse inducing robust lung inflammation [20,21]. Anti-IL-1α antibody (clone AL-161; e-Biosciences, San Diego, USA) and anti-IL-1β antibody (clone B-122; e-Biosciences) were injected into the peritoneal cavity at a dose of 80 μg/mouse 1 hour before silica instillation [5].

### Broncho-alveolar lavage (BAL) and alveolar cell number

Mice were sacrificed with an intraperitoneal injection of sodium pentobarbital (20 mg/mice) and bronchoalveolar lavage was performed by cannulating the trachea and lavaging the lung 4× with 1ml of NaCl 0.9%. The broncho-alveolar lavage fluid (BALF) was centrifuged at 281g, 10 min, 4°C (Centrifuge 5804R, Eppendorf, Hamburg, Germany). Cells recovered in BAL were counted then fixed in 1.25% paraformaldehyde and analyzed with FACS calibur (BD Biosciences) using FlowJo software. Neutrophils were identified by labelling with anti-GR1-PE (clone RB6-8C5, BD Biosciences, Erembodegem, Belgium) and their number calculated in function of total BAL cells counted with a Burker cell chamber. Fc receptors were blocked with anti-CD16/CD32 (clone 2.4G2, BD Biosciences) to reduce nonspecific binding.

### Lung histopathology

Lungs were lavaged and perfused with 0.9% NaCl and superior left lung lobe was fixed in 3.6% formaldehyde solution (Sigma-Aldrich) during one night. Paraffin-embedded 5-μm sections were stained with hematoxylin and eosin. Images were acquired with a slide scanner SCN400 and analyzed with Digital Image Hub (Leica Microsystems, Diegem, BE).

### RNA extraction and quantification

Total RNA extraction and quantification by qRT-PCR were performed as described [86]. Sequences of interest were amplified by PCR using the following forward primers (Invitrogen): CGG CTA CCA CAT CCA AGC AA (mouse Rn18s ), GAC GGA CCC CAA AAG ATG AAG (mouse Il1b ), GGA CTT CTC AAG ATC ATG GCT ACT T (mouse Cxcr2 ), TTG AAG ACC TAA AGA ACT GTT ACA GTG AA (mouse Il1a ), GGA AAA GAC CAA GAG CAA GAC C (mouse Il33), TTT TGT CCA CAT GCC CTG C (mouse Hmgb1 ), and reverse primers: ATA CGC TAT TGG AGC TGG ATT ACC (mouse Rn18s), CTC TTC GTT GAT GTG CTG CTG TG (mouse Il1b ), TAG TAG AGG TGT TTG CTG AAG ACG A (mouse Cxcr2), GCC ATA GCT TGC ATC ATA GAA GG (mouse Il1a), TTC TTC CCA TCC ACA CCG TC (mouse Il33), CTA ATA GTC CCA CGG TGT GAC AGT (mouse Hmgb1).

### Cell purification

Mice treated with silica (2,5 mg/mouse) or not were sacrificed after 3 hours by intramuscular injection of 60 mg sodium pentobarbital. Lungs were perfused via the right heart ventricle with sterile NaCl 0.9%. One ml of enzyme mix containing 20 mg of pronase (Sigma-Aldrich) and 1mg of dnase (Worthington Biochemical Corporation, Lakewood, USA) in HBSS (Invitrogen; Merelbeke; Belgium) with 1% of antibiotic antimycotic (AA) (fungizone (25 μg/mL), penicillin–streptomycin (10000 U and 10000 μg/mL); Invitrogen) were infused in cannulated trachea. After 20 minutes, lungs were excised and placed into a tube chilled on ice with fetal bovine serum (FBS) (Invitrogen). Collected lungs were then crushed by repeated aspiration and expulsion in a 20 ml seringue and passed on a 70 μM filter. Structural cells were purified based on their absence of CD45 expression by using magnetic cell separation (MACS; Miltenyi Biotec) according to the manufacturer's protocol; T lymphocytes based on their CD5 expression; B lymphocytes based on their B220 expression; dendritic cells based on their absence of F480 and the presence of CD11c expression; and alveolar macrophages based on their F480 expression. Alveolar macrophages were also obtained from lung cell suspensions grown in 75cm^2^ tissue culture flask in DMEM (Invitrogen) supplemented with 10% FBS and 1% of AA, detached using trypsin (Invitrogen) and purified based on their CD45 expression by using magnetic cell separation (MACS) according to the manufacturer's protocol.

### Pro-IL-1β and IL-1α content in lung cells

For ELISA measurement of intracellular or released pro-IL-1β or IL-1α, fresh lung cells purified from naïve or silica treated mice, primary cultured macrophages, J774, MLg or LA4 (10^6^ cells/well in 96-well plate) were exposed overnight with LPS (0,1μg/ml; Enzo Life Sciences, Antwerpen, Belgium), DQ12, mouse recombinant IL-1α, IL-33, IL-1β, TNF-α (R&D systems, Minneapolis, USA) or HMGB1 (chemokine, non-oxydable and cytokine form; HMGBiotech, Milano, Italia) when necessary. For intracellular cytokine measurement, cell pellets were lysed by addition of 100 μl of triton X-100 0,1%. The ex vivo released of IL-1α were quantified by ELISA in the supernatants of cells purified from silica-treated lungs maintained in culture during 24 hours in 100 μl of DMEM. For *in vitro* IL-1α release in response to particles, fresh alveolar F4/80^+^ macrophages (10^6^ cells/well in 96-well plate) or J774 cells (0.4 10^6^ cells/well in 96-well plate) were incubated overnight with particles dispersed in 100 μl of DMEM and IL-1α was measured in the supernatant.

For western blot analysis, cells were lysed on ice in 200 μl Triton lysis buffer (TLB) (1% Triton, 25mM Tris pH 7.4, 150mM NaCl + anti-protease tablets from Roche Applied Science at 2 mg/ml). Protocol for Western blot is described elsewhere [84].

### Enzyme-linked immunosorbent assays (ELISA)

ELISA kits were used to measure IL-1β, IL-1α, IL-33 (DY401, R&D Systems, Wiesbaden-Nordenstadt, Germany), HMGB1 (Cedarlane Laboratories USA Inc., Burlington, USA) and pro-IL-1β (e-Biosciences). Assays were run according to the manufacturer's protocols with a detection limit of 5 pg/ml for IL-1β, IL-1α, IL-33, 0,2 ng/ml for HMGB1 and 25 pg/ml for pro-IL-1β

### Statistics

Results were analyzed by *t*-test. Statistical significance was considered at P < 0.05.

## Additional file

Additional file 1
**Figure S1.** Levels of (a) HMGB1, (b) IL-1β and proteins (c) in BAL fluid collected at different time points after silica (crystalline DQ12, 2.5 mg) or not (control). Pulmonary expression of (d) pro-IL-1α, (e) pro-IL-33 and (f) HMGB1 quantified by qRT-PCR at different time points after instillation of silica or not. Values are means ± SEM of 3 to 8 animals. *p <0.05, **p < 0.01 and ***p < 0.001 denote significant difference between animals treated with silica or not; ns, denotes no significant difference. P-values are estimated by t-test. **Figure S2.** (a) Intracellular levels of pro-IL-1β in primary cultured alveolar macrophages exposed overnight to LPS (0.1 μg/ml) or recombinant TNF-α. n = 3 to 5. (b) Western blot analysis of intracellular pro-IL-1β and β-actin in primary cultured lung macrophages exposed during 18 hours to LPS (0.1 μg/ml) or recombinant TNF-α. Values are means ± SEM. **p < 0.01 and ***p < 0.001 denotes significant difference between cells in DMEM and cells exposed to LPS or recombinant TNF-α. P-values are estimated by t-test.
