# Supplementary material for: The alarmin IL-1α is a master cytokine in acute lung inflammation induced by silica micro- and nanoparticles
Source: Part Fibre Toxicol. 2014 Dec 13;11:69. doi: 10.1186/s12989-014-0069-x (PMC4279463; doi:10.1186/s12989-014-0069-x)

The alarmin IL-1α is a master cytokine in acute lung inflammation induced by silica micro- and nanoparticles

Virginie Rabolli, et co-authors

Supplemental legends

Supplemental figure 1 : Levels of **(a)** HMGB1, **(b)** IL-1β and proteins **(c)** in BAL fluid collected at different time points after silica (Crystalline DQ12, 2.5 mg) or not (control). Pulmonary expression of **(d)** pro-IL-1α, **(e)** pro-IL-33 and **(f)** HMGB1 quantified by qRT-PCR at different time points after instillation of silica or not. Values are means ± SEM of 3 to 8 animals. *p < 0.05, **p < 0.01 and ***p < 0.001 denote significant difference between animals treated with silica or not; ns, denotes no significant difference. P-values are estimated by t-test.

Supplemental figure 2 : (a) Intracellular levels of pro-IL-1β in primary cultured alveolar macrophages exposed overnight to LPS (0.1 µg/ml) or recombinant TN-α. n = 3 to 5. **(b)** Western blot analysis of intracellular pro-IL-1β and β-actin in primary cultured lung macrophages exposed during 18 hours to LPS (0.1 µg/ml) or recombinant TNF-α. Values are means ± SEM. **p < 0.01 and ***p < 0.001 denotes significant difference between cells in DMEM and cells exposed to LPS or recombinant TNF-α. P-values are estimated by t-test.

Supplemental Figure 1

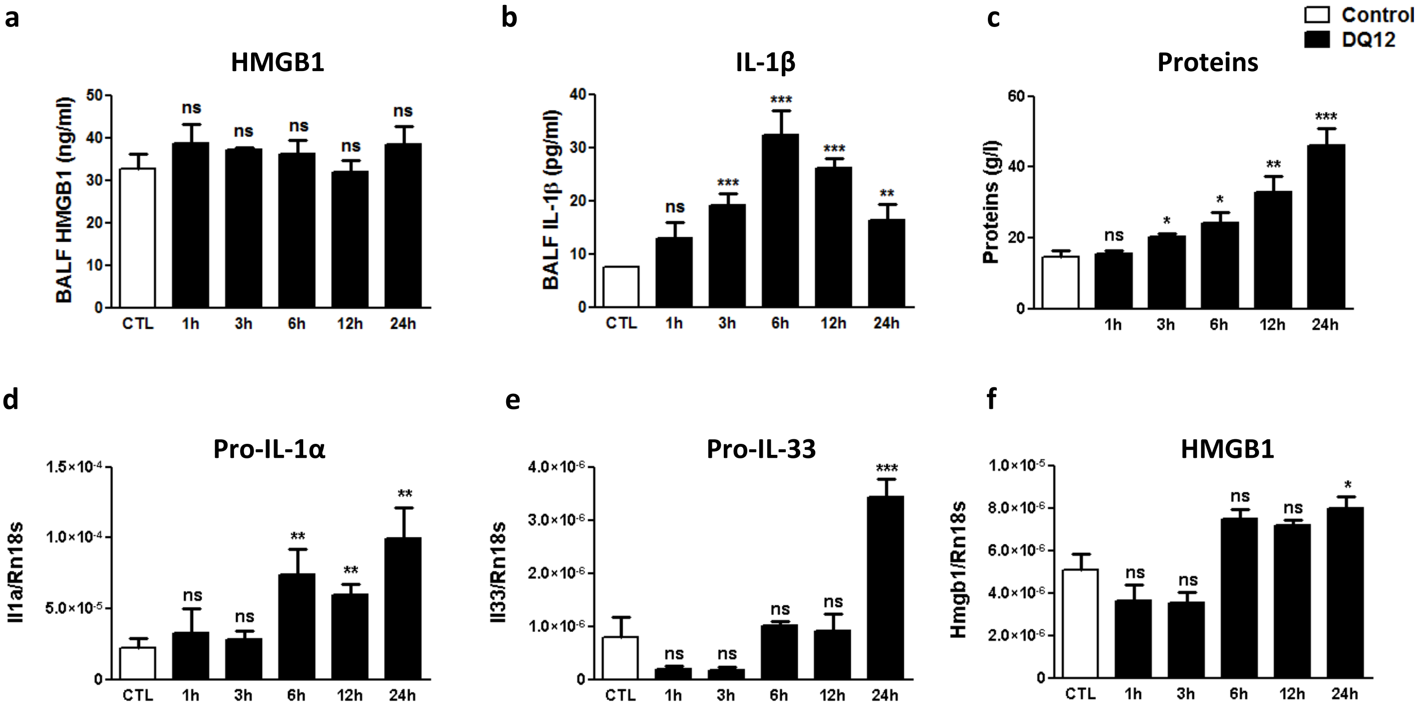

Supplemental Figure 2

a

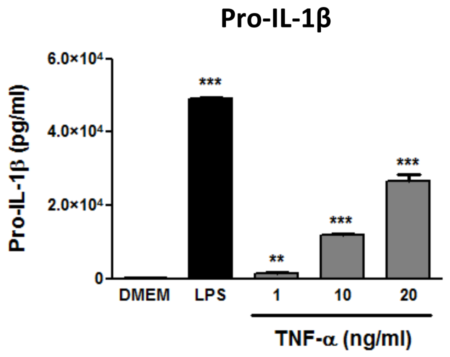

b

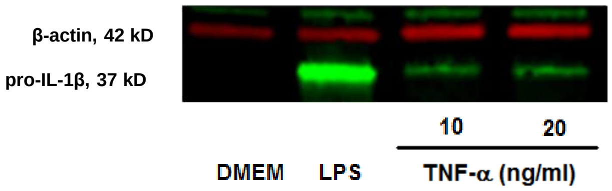

Supplement: Additional file 1 — Figure S1. Levels of (a) HMGB1, (b) IL-1β and proteins (c) in BAL fluid collected at different time points after silica (crystalline DQ12, 2.5 mg) or not (control). Pulmonary expression of (d) pro-IL-1α, (e) pro-IL-33 and (f) HMGB1 quantified by qRT-PCR at different time points after instillation of silica or not. Values are means ± SEM of 3 to 8 animals. *p <0.05, **p < 0.01 and ***p < 0.001 denote significant difference between animals treated with silica or not; ns, denotes no significant difference. P-values are estimated by t-test. Figure S2. (a) Intracellular levels of pro-IL-1β in primary cultured alveolar macrophages exposed overnight to LPS (0.1 μg/ml) or recombinant TNF-α. n = 3 to 5. (b) Western blot analysis of intracellular pro-IL-1β and β-actin in primary cultured lung macrophages exposed during 18 hours to LPS (0.1 μg/ml) or recombinant TNF-α. Values are means ± SEM. **p < 0.01 and ***p < 0.001 denotes significant difference between cells in DMEM and cells exposed to LPS or recombinant TNF-α. P-values are estimated by t-test. [file 12989_2014_69_MOESM1_ESM.pdf]
